# Reliability of High-resolution Gadolinium-enhanced MR Cisternography and Gasket-seal Technique for Management of Anterior Skull Base Defects

**DOI:** 10.1007/s00062-023-01339-2

**Published:** 2023-09-01

**Authors:** Mukesch Johannes Shah, Jürgen Beck, Stephan Meckel, Horst Urbach, Ikram Eda Duman, Manuel Christoph Ketterer, Tanja Hildenbrand

**Affiliations:** 1 Department of Neurosurgery, Medical Center, University of Freiburg, Breisacher Straße 64, 79106 Freiburg, Germany; 2 Institute for Diagnostic and Interventional Neuroradiology, RKH Klinikum Ludwigsburg, Posilipostraße 4, 71640 Ludwigsburg, Germany; 3 Department of Neuroradiology, Medical Center, University of Freiburg, Breisacher Straße 64, 79106 Freiburg, Germany; 4 Department of Oto-Rhino-Laryngology, Medical Center, University of Freiburg, Killianstr. 5, 79106 Freiburg, Germany

**Keywords:** CT cisternography, Cerebrospinal fluid leak, Anterior skull base reconstruction, Spontaneous cerebrospinal fluid rhinorrhea, CS T1 SPACE sequence

## Abstract

**Purpose:**

Precise preoperative localization of anterior skull base defects is important to plan surgical access, increase the success rate and reduce complications. A stable closure of the defect is vital to prevent recurrence of cerebrospinal fluid (CSF) rhinorrhea. The purpose of this retrospective case series was to evaluate the reliability of a new high-resolution gadolinium-enhanced compressed-sensing SPACE technique (CS T1 SPACE) for magnetic resonance (MR) cisternography to detect cerebrospinal fluid leaks of the anterior skull base and to assess the long-term success rate of the gasket-seal technique for closure of skull base defects.

**Method:**

All patients with spontaneous or postoperative cerebrospinal fluid rhinorrhea and defects of the anterior skull base presenting to the Departments of Otorhinolaryngology and Neurosurgery between 2019 and 2020, receiving a computed tomography (CT) cisternography and MR cisternography (on a 3T whole-body MR scanner using a 64-channel head and neck coil) with CS T1 SPACE sequence and closure of the defect with the gasket-seal technique, were enrolled in the study. For the cisternography, iodinated contrast agent (15 ml Solutrast 250 M®), saline (4 mL) mixed with a 0.5 mL of gadoteridol was injected into the lumbar subarachnoid space.

**Results:**

A total of four patients were included in the study and MR cisternography with CS T1 SPACE sequence was able to precisely localize CSF leaks in all patients. The imaging results correlated with intraoperative findings. All defects could be successfully closed with the gasket-seal technique. The mean follow-up was 35.25 months (range 33–37 months).

**Conclusion:**

MR cisternography with CS T1 SPACE sequence could be a promising technique for precise localization of CSF leaks and the gasket-seal technique resulted in good closure of the CSF fistula in this case series.

## Introduction

Trauma or iatrogenic injury during surgery of the inner nose and paranasal sinuses are the main causes for defects of the anterior skull base with cerebrospinal fluid (CSF) rhinorrhea. Diagnosis and localization of these defects is usually without difficulty during the causative surgical procedure, or in cases of trauma, on conventional computed tomography (CT) scans. The same holds true for defects after endonasal endoscopic skull base surgery. Localization of the defect is usually more challenging in cases of spontaneous CSF rhinorrhea. Persistence of the lateral craniopharyngeal canal (also known as Sternberg’s canal; for defects within the sphenoid sinus) and idiopathic intracranial hypertension are discussed as possible causes [1–3]. Patients are typically middle-aged overweight women. Additional symptoms resembling those of hydrocephalus may be present, e.g., headache, impaired vision and balance.

Untreated CSF leaks bear a high risk for meningitis and other intracranial complications [4–6]. The precise preoperative localization of the defect facilitates detailed planning of surgical access, increases the success rate and reduces complications [7]. CT cisternography (CTC) is an established method to localize dural defects [8, 9]. Intrathecal administration of iodinated contrast medium can cause severe allergic reactions and seizures. Intrathecal gadolinium-enhanced MR cisternography (MRC) is a new method to localize CSF fistulas. Intrathecal administration of gadolinium is off-label and the patients need to consent to this. Studies have reported its safety and success in locating CSF leaks [10]. T1 SPACE sequence with compressed-sensing method (CS T1 SPACE) for 3D imaging, a MR technical innovation for the 3 T scanner, has been introduced in our hospital, offering high spatial resolution in submillimeter ranges with almost isotropic resolution (voxel size 0.5 × 0.5 × 0.6 mm) and with acceptable scanning times (6:50 min). The high signal-to-noise ratio and good fat suppression of this sequence promote visualization of very small contrast leakages from the intracranial CSF space through the bony skull base. Clear superiority of this sequence compared to traditional CTC in visualizing CSF leaks of the skull base has been shown in a pilot study on the technical evaluation of this sequence in seven patients with CSF rhinorrhea [11].

There are different surgical techniques for the closure of dural defects. Typically, different layers of autologous or alloplastic material are positioned into or onto the defect as underlay and/or overlay. Materials used are synthetic materials, free tissue grafts and local pedicled flaps alone or in combination. The gasket-seal technique was first described by Leng and Schwartz [12]. The principle is to create a watertight sealing ring. First, the defect is closed with an underlay, e.g., with alloplastic material, such as Tachosil® (Takeda Pharma Vertrieb GmbH & Co. KG, Berlin, Germany) oder DuraGen® (Integra LifeSciences Coporation, Princeton, NJ, USA) or autologous tissue, such as the fascia lata, intracranially between the bony skull base and dura. An additional layer of this alloplastic or autologous material is placed extracranially onto the skull base as an overlay, clearly overlapping the defect. A piece of autologous bone or alloplastic bone graft material cut to the size of the defect is pushed into the defect, so that the overlay material is circumferentially wedged between the bony edge of the defect and the bone graft, creating a sealing ring. This technique facilitates a mechanically stable and watertight closure of the defect.

The aim of this retrospective case series was to assess the reliability of the intrathecal gadolinium-enhanced MRC with CS T1 SPACE sequence in the preoperative visualization of dural defects of the anterior skull base by comparing the preoperative imaging results with intraoperative findings. In addition, we planned to evaluate the long-term success rate of the gasket-seal technique for surgical CSF leak closure.

## Material and Methods

All patients with spontaneous or postoperative defects of the anterior skull base presenting to the departments of otorhinolaryngology or neurosurgery between December 2019 and 2020, receiving a CTC and MRC with CS T1 SPACE sequence in the department of neuroradiology and closure of the defect with the gasket-seal technique, were enrolled in the case series. Patients under 18 years of age at first diagnosis and without written informed consent for data collection and evaluation were excluded.

The imaging technique of MRC has been described and the technical feasibility has been evaluated in a pilot study [11] and was performed in this way on each patient. After lumbar intrathecal injection of iodine contrast agent (15 ml Solutrast 250 M®, Bracco Altana Pharma, Konstanz, Germany), saline (4 mL) mixed with a 0.5 mL of gadoteridol (ProHance, Bracco Diagnostics, Princeton, NJ, USA) was injected into the subarachnoid space. The patients were positioned prone in the 30–40° Trendelenburg position for 10–20 min after withdrawal of the needle to maximize the potential for contrast agent accumulation in the intracranial basal subarachnoid cisterns. Immediately after, with the patient in a prone position, CT cisternography images and 3h after the injection while the patient was kept in prone position, MR cisternography images on a 3T whole-body MR scanner (Magnetom Prisma, Siemens Healthcare GmbH, Erlangen, Germany) using a 64-channel head and neck coil were obtained with the patient in a supine position.

CTC is performed on a 64-section CT scanner (Somatom CT Definition AS, Siemens Healthcare GmbH): section acquisition, 2 × 0.6 × 64, by means of a z-flying focal spot; gantry rotation, 0.5 s; tube voltage, 80 kV; tube current-time product, 236 mA/pitch; sharp kernel (B60) reconstructed images (field of view, 100 mm; section thickness, 0.6 mm; increment, 0.4 mm). Bone and soft tissue algorithms were applied to enhance bone and contrast media details.

For MRC, a highly accelerated CS T1 SPACE sequence was applied on a 3T whole-body MR scanner (Magnetom Prisma, Siemens) using a 64-channel head and neck coil. The sequence uses a Poisson-disc variable density acquisition with elliptical *k*-space coverage. Image reconstruction was done by combination of CS with L1 norm-based regularization in the wavelet domain and parallel imaging (number of iterations 20; regularization parameter 0.0013). A whole-head sagittal T1-weighted CS SPACE protocol (TR/TE, 800/5.1 ms; field of view, 210 × 210 mm^2^; 256 slices; section thickness, 0.60 mm; matrix, 384 × 384; fat saturation; pixel band width 450 Hz/px; echo-train length, 0; total scan time, 6:50 min) with a *k*-space undersampling factor of 0.22 was implemented, which yields an approximate fivefold acceleration compared with full *k*-space sampling. The CS SPACE T1 sequence had an almost isotropic 3D resolution (0.5 × 0.5 × 0.6 mm^3^) [3, 11].

In addition, a sagittal MPRAGE (voxel: 1 × 1 × 1 mm^3^) was performed in all patients.

The patients of this case series were included in the technical pilot study [11]. The gasket-seal technique has been described in detail elsewhere and is briefly described in the introduction section [12].

CTC and MRC as well as duraplasty were indicated by a neurosurgeon or otolaryngologist with experience in skull base surgery, based on the patient’s clinical findings suspicious for a cranial CSF leak.

The analysis is based on statistical data from the department of neuroradiology, the patients’ medical records, surgical notes and digitalized examination results.

The primary objectives of the study were to compare the preoperative radiological localization of the skull base defect to intraoperative findings and to evaluate the long-term success rate of the gasket-seal technique for CSF leak closure.

Secondary objectives were to record complications of CTC, MRC and duraplasty and to observe possible influencing factors, such as age, sex, smoking and comorbidities (e.g., diabetes, hypertension).

The study was conducted according to national regulations and the 1964 declaration of Helsinki and its later amendments as well as the Note for guidance on good clinical practise (CPMP/ICH/135/95) (GCP) from 17 January 1997 [13]. The study was approved by the local ethics committee (Vote number: 398/20). The participants were given an information sheet about the study and gave written informed consent. They were also informed about their rights according to current data protection regulations and gave written consent for the collection, analysis and storage of their personal data.

## Results

During the study period 7 patients (5 females, 2 males, mean age 53.1 years) underwent combined CTC/MTC studies. Of the patients three had to be excluded from the study because two had a CSF leak in the mastoid bone and one had a leak in the lateral aspect of the sphenoid sinus that was not closed with the seal technique. Finally, four patients were included in the study (4 females, mean age 57.8 years). Epidemiological data of the patients are summarized in Table 1. All but one patient suffered from spontaneous CSF rhinorrhea. This patient had a history of partial resection and radiotherapy for pituitary macroadenoma. All patients showed an elevated body mass index (BMI) of > 25, which is defined as overweight or obese, one patient suffered from arterial hypertension and type 2 diabetes and one patient was diagnosed with underlying idiopathic intracranial hypertension (IIH) during the follow-up period. Another patient had a history of right breast cancer and synchronous multiple myeloma without involvement or prior surgery of the anterior skull base. The remaining patient had no relevant comorbidities.

**Table 1:** Epidemiological data of study participants

Patient no.	Age (years)	BMI	Medical history
1	55	28	None
2	60	33.5	Post-deep vein thrombosis
3	69	25.2	Multiple myeloma, first diagnosed 2016; synchronous breast cancer, first diagnosed 2016
4	47	32.6	Post-partial resection of pituitary macroadenoma 2006/2008, post-radiotherapy 2008, post-meningitis (2006, 2019). Hypertension, type 2 diabetes

The results of the imaging studies, beta 2‑transferrin results and intraoperative findings are summarized in Table 2. All patients had fluorescein injected intrathecally preoperatively (0.1 ml/10 kg body weight of 1% sodium fluorescein solution, off-label) to improve intraoperative localization of the dural defect. All patients received a lumbar drain intraoperatively that was left in place for 3–5 days postoperatively. No complications occurred during and after cisternography or dural repair. The mean follow-up was 35.25 months (range 33–37 months). The primary success rate of the gasket-seal technique was 100%, 2 patients were not primarily managed with the gasket-seal technique, 1 patient (patient 1) did not show CSF flow or a bony defect of the skull base during the first surgery and the cribriform plate was sealed with Tachosil®. The defect of another patient (patient 3) was primarily closed with an overlay of DuraGen® and fat. Both patients showed recurrence of CSF rhinorrhea.

**Table 2:** Diagnostic and intraoperative findings. For patients with multiple surgeries: (1) first surgery/preoperatively, (2) second surgery/preoperatively

Pt. no	Cause of cerebrospinal fluid rhinorrhea	Beta 2‑transferrin	Localization of defect	Identified on CTC	Identified on MRC	Confirmation intraoperatively	Material for CSF leak closure
1	Idiopathic/ idiopathic intracranial hypertension	Positive	Left cribriform plate	(1) Yes/(2) No	(1) No MRC performed (2) Yes (foramen caecum anterior to crista galli)	(1) No CSF flow intraoperatively (2) Yes	Tutopatch®, LactoSorb®, mucosa
2	Idiopathic	Positive	Left cribriform plate	Yes	Yes (left olfactory groove)	Yes	DuraGen®, Resorba® X
3	Idiopathic	Positive	Posterior roof of ethmoid	(1) No, suspected leak at roof of sphenoid sinus with bony wall thinning /(2) No	(1)/(2) Yes (right posterior ethmoidal roof)	Yes	(1) DuraGen®, fat (2) Fat intracranially, Tutopatch®, LactoSorb®
4	Postoperative/ Post-radiotherapy for pituitary macroadenoma	Not evaluated	Sphenoid sinus	Yes, but only suspected at right anterior sphenoid sinus	Yes (right sphenoid sinus, intrasellar pouch with small channel to sphenoid sinus)	Yes	Fat, LactoSorb®, Adherus AutoSpray

*Adherus AutoSpray* Stryker, Kalamazoo, MI, USA, *LactoSorb®* Zimmer Biomed, Zug, Switzerland; *Resorba® X* Resorba Medical GmbH, Nürnberg, Germany; *Tutopatch®* RTI Surgical, Greenville, NC, USA

In two patients CTC failed to detect the exact site of the CSF leak. In contrast, MRC was able to identify all CSF leaks and the results correlated with intraoperative findings (Table 2).

### Case Descriptions

#### Patient 1

**Fig. 1:**
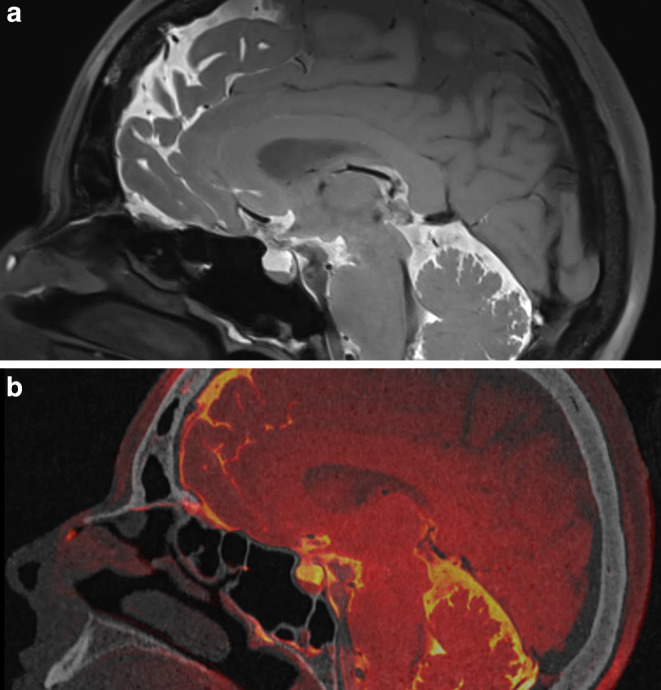
Sagittal section of (**a**) intrathecal gadolinium enhanced magnetic resonance cisternography (MRC)  and (**b**) fusion images of MRC and contrast enhanced computed tomography cisternography (CTC) of patient 1. Both images show clear depiction of CSF leak at left cribriform plate anterior to crista galli

**Fig. 2:**
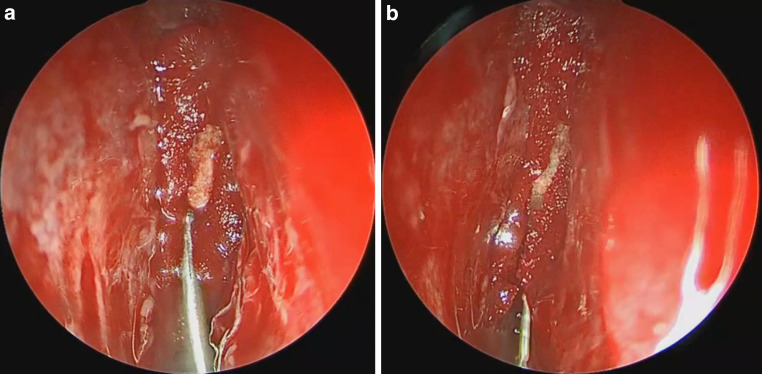
Gasket-seal technique. Endoscopic view of left cribriform plate after placement of Tutopatch® as underlay and overlay and LactoSorb® countersunk into the defect. The overlay material is pushed to the edges of the defect to act as a sealing ring

A 55-year-old female presented with a history of recurrent left-sided clear nasal discharge. At first presentation, conventional CTC showed an equivocal result with a suspected leak at the left cribriform plate. Functional endoscopic sinus surgery and blue light endoscopy were performed after intrathecal fluorescein injection to localize the site of the leak, but no intranasal fluorescein could be detected. The cribriform plate was sealed with Tachosil® and 1 year later the patient presented with recurrent left-sided clear nasal discharge and persistent mild headaches while sitting or standing. The CTC failed to reveal a CSF leak and CS SPACE MRC images showed a fine tubular leak from the anterior foramen caecum to the crista galli into the left nasal cavity (Fig. 1a, b). Fused images of CTC and CS SPACE MRC allowed a better localization of the CSF leak in relation to the bony structures of the anterior skull base, pinpointing the exact site were the fistula crossed the osseous skull base (Fig. 1b). The site correlated with the patient’s intraoperative findings. The defect was closed with Tutopatch® (underlay + overlay), LactoSorb® and mucosa (overlay) (Fig. 2a, b). There was no recurrence of CSF rhinorrhea 35 months postoperatively. Postoperatively, the patient was diagnosed with IIH with typical features of empty sella, optic sheath widening and papilledema.

#### Patient 2

**Fig. 3:**
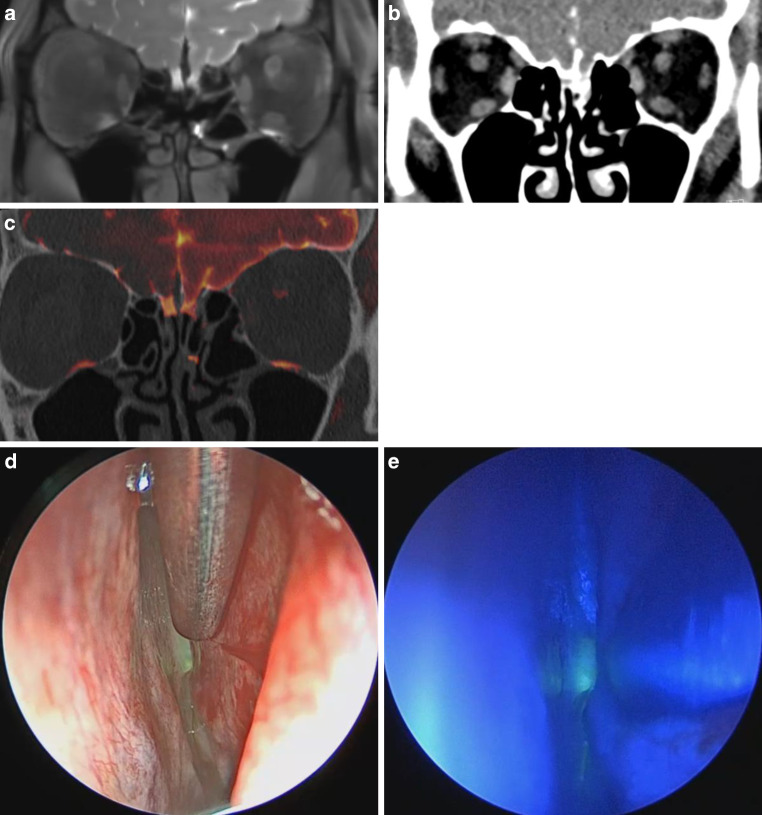
MRC and CTC of patient 2. **a** Coronal section of MRC with evidence of CSF leak located in left olfactory groove. **b** Coronal section of CTC with suspected CSF leak but inferior quality compared to MRC and fusion images. **c** Coronal section of fusion images of MRC and CTC showing the exact location of the CSF leak. **d**, **e** Endoscopic images: (**d**) intraoperative correlation with CSF leak in left olfactory groove after intrathecal fluorescein application and (**e**) with connection of blue light filter

The 60-year-old female patient presented with a history of intermittent left-sided clear rhinorrhea that first occurred in 2018, but subsided spontaneously. After recurrence of rhinorrhea, beta 2‑transferrin in nasal secretions was positive and she was referred to CTC and MRC. CTC showed a CSF leak in the left olfactory groove that was confirmed on MRC. The fusion images of CTC and CS SPACE MRC facilitated exact localization of the defect, which correlated with intraoperative findings (Fig. 3a, b, c, d and e). The defect was closed with DuraGen® (underlay + overlay) and Resorba® X. The postoperative course was unremarkable and there was no recurrence of CSF rhinorrhea during the following 33 months.

#### Patient 3

A 69-year-old woman had a history of right breast cancer and synchronous multiple myeloma without involvement or prior surgery of the anterior skull base. The only surgical intervention in the head and neck region was maxillary sinus surgery through a transoral approach 9 months earlier. The patient suffered from an upper respiratory tract infection 2 weeks prior to presentation and 1 week after this, she developed clear right-sided rhinorrhea. Beta‑2 transferrin was positive.

**Fig. 4:**
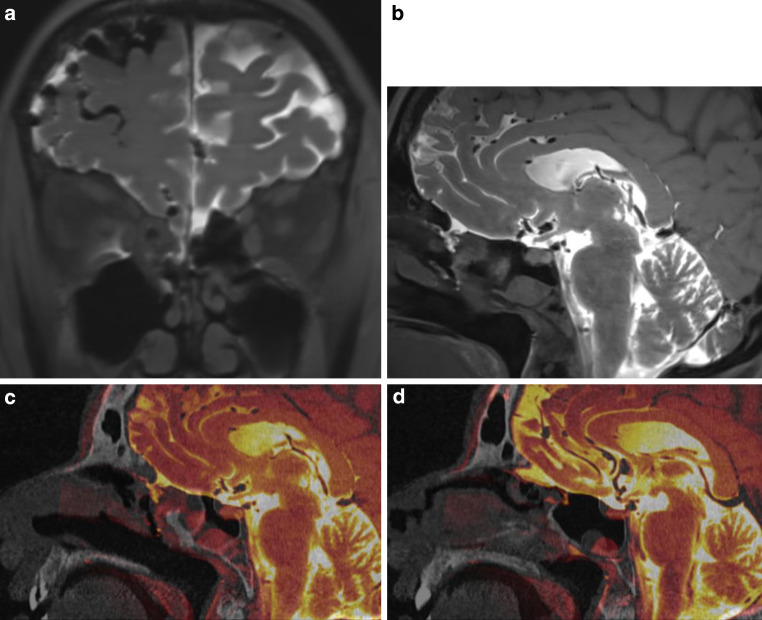
Coronal (**a**) and sagittal (**b**) section of MRC and sagittal sections of fusion images of MRC and CTC (**c**, **d**) of patient 3. CSF leak is localized at right posterior cribriform plate. Secondary CSF flow along anterior wall of sphenoid sinus (**d**)

Combined CTC and MRC was performed. CTC images showed a suspicious area of bone thinning at the roof of sphenoid sinus with no clear CSF fistula despite an anterior fluid level of contrast medium in the inferolateral recess of the right sphenoid sinus with the patient in prone position on CTC. The CS SPACE MRC images were able to demonstrate a leakage of contrast medium anterior to the sphenoid sinus at the right cribriform plate into the posterior ethmoidal air cells. Moreover, progressive extracranial contrast medium accumulation with an air-fluid level in the right sphenoid sinus and posterior ethmoid was shown related to the supine position during MRI. For CSF fistula repair, endonasal endoscopic duraplasty was performed. CSF flow and exposed dura were visible in the posterior ethmoid adjacent to the anterior wall of the sphenoid sinus, but CSF flow stopped during surgery. The area of exposed dura was covered with an onlay of DuraGen® and fat. No CSF leak was seen in the sphenoid sinus. Less than 1 month after surgery, recurrent CSF rhinorrhea was noted. Combined CTC and MRC was repeated. Again, CTC images alone failed to reveal the exact site of CSF leak with a suspected area of bone lucency at the anterior cribriform plate and diffuse accumulation of contrast medium in the right sphenoid sinus and ethmoid. The CS SPACE MRC images showed the same site of leakage anterior to the sphenoid sinus at the roof of the right ethmoid into the posterior ethmoidal air cells (Fig. 4a, b). Fusion image of CTC and CS SPACE MRC accurately pointed to the small site of CSF leak (Fig. 4c). A second endonasal endoscopic surgical repair procedure was performed. The site of leakage was confirmed and closed with fat intracranially, Tutopatch® as overlay and Lactosorb® and 36 months postoperatively she showed no recurrence of CSF rhinorrhea.

#### Patient 4

**Fig. 5:**
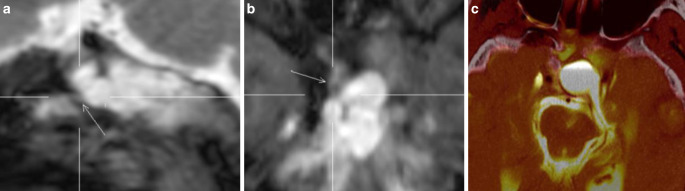
Sagittal (**a**) and coronal (**b**) section of MRC and fusion images (**c**) of patient 4 showing a CSF leak from an intrasellar pouch with a small channel to sphenoid sinus (*white arrow*)

A 47-year-old female had undergone transsphenoidal partial resection in 2006 and endoscopic transsphenoidal pituitary surgery and concomitant stereotactic radiotherapy in 2008 for pituitary macroadenoma with extensive erosion of the skull base. No CSF rhinorrhea occurred postoperatively. She developed severe meningitis after surgery and radiotherapy, which was treated with ceftriaxone for 2 weeks. At this time, no investigation for CSF leak was performed but 13 years later, the patient was readmitted with headache, clear rhinorrhea and recurrent pneumococcal meningitis. Combined CTC and MRC were performed. A bony defect at the resection site in the right sphenoid sinus and along the cavernous sinus with accumulation of contrast medium in the sphenoid sinus was seen on CTC. CS SPACE MRC better delineated the leakage of contrast medium from the resection site into the right sphenoid sinus (Fig. 5a, b). Fused images of CTC and MRC were able to improve visualization of the exact site of CSF leak (Fig. 5c). The leak was confirmed intraoperatively and closed with fat, LactoSorb® and Adherus. There has been no recurrence during the 37 months follow-up.

## Discussion

Preoperative localization of dural defects is crucial for successful repair, especially in cases of spontaneous CSF rhinorrhea.

High-resolution gadolinium-enhanced MRC is a promising technique that can enable direct and sensitive visualization of the site of spontaneous, posttraumatic or postoperative CSF leaks. Gadolinium-enhanced MRC has certain advantages over CTC. The high viscosity of iodinated contrast agent that is used for CTC may impair its free distribution in the subarachnoid space and its passage into slowly flowing CSF fistulas. In contrast, gadolinium distributes readily in the subarachnoid space [10]. In CTC it can also be difficult to differentiate CSF leakage from adjacent bony structures, especially in areas of thin bone such as the cribriform plate, as CT density values are similar to those of the contrast agent diluted with CSF. Further advantages of MRC are absence of radiation exposure and high soft tissue resolution. Several studies have reported the safe off-label use of intrathecally administered gadopentetate dimeglumine for MRC [14–17]. Neurotoxicity has been reported after intrathecal administration of high doses of gadolinium [18, 19]. It is a rare but potentially serious complication that can manifest as various neurological symptoms. Low doses of intrathecal gadopentetate dimeglumine that are adequate for diagnostic enhancement of the subarachnoid space of humans for MRI do not show clinical evidence of gross physical or neurologic adverse events [20]. Nevertheless, physicians should carefully consider the risks and benefits of this procedure in each individual patient and monitor for any signs of neurotoxicity. There are additional advantages of the new high-resolution 3D CS SPACE T1 sequence for MRC after intrathecal gadolinium administration. It provides a global view of the entire skull base, meninges, and brain. Due to the high signal-to-noise ratio and fat suppression, which increase the contrast between hypointense bony or aerate cell structures, hyperintense CSF and isointense soft tissue, the sequence seems more sensitive to detect CSF leaks. Further advantages relate to the high spatial detail with 3D capabilities (almost isotropic submillimeter resolution) providing an excellent contrast-to-noise ratio post-gadolinium. Fusion of CTC and MRC images enables exact localization of CSF leaks in relation to bony landmarks of the anterior skull base.

In contrast to MRC, CTC failed to detect the exact site of the bony defect and CSF leak in two of our cases (patients 1 and 3). All leaks detected with MRC were confirmed intraoperatively. In patient 1, only CTC was performed prior to the first surgery, as MRC was not available at that time. A CSF leak at the left cribriform plate was only suspected by CTC. No bony defect or CSF flow was visible during surgery, so the exact localization of the defect was not possible. Prior to the second surgery, CTC again failed to detect the defect and only CS SPACE MRC images showed the fine tubular leak from the anterior foramen caecum to the crista galli into the left nasal cavity, which was confirmed during surgery and successfully closed with the gasket-seal technique. In patient 3, the suspected site of the CSF leak on CTC did not correlate with the actual site, which could only be detected on MRC and this correlated with the intraoperative finding. Related to differences in patient positioning during MRC (supine) and CTC (prone), the 3D CS SPACE cisternography images show a more posterior contrast media accumulation (e.g. posterior ethmoid/sphenoid sinus from leaks at the anterior skull base), whereas CTC shows anterior contrast media accumulation towards the nasal cavity. This has to be considered when interpreting the imaging results, as it could lead to misinterpretation. In patient 3, due to the supine position during MRI, the contrast media secondarily accumulated in the right sphenoid sinus, which led to misinterpretation. Only CS SPACE cisternography MRI images revealed the exact site of the CSF leak at the ethmoidal roof.

Four patients met the inclusion criteria for the current study. All the patients with spontaneous CSF leaks were middle-aged overweight women, which is known to be the typical patient characteristic. One patient was diagnosed with idiopathic intracranial hypertension (IIH) postoperatively, which is discussed as a possible cause for spontaneous CSF leaks [1–3]. She showed empty sella and widening of optic sheath on MRI and CSF pressure of 33 cm H_2_O. In addition, she showed typical clinical signs of IIH, i.e., headaches and balance problems. In a study by Rupa et al. the majority of patients with spontaneous CSF rhinorrhea showed MRI features of IIH, e.g., empty sella, flattened posterior globe, enlarged Meckel’s cave [21]. It is reasonable to check for signs of IIH in patients with spontaneous CSF rhinorrhea with typical patient characteristics and clinical signs and initiate treatment to prevent recurrence postoperatively [1].

Defects of all patients closed with the gasket-seal technique showed no recurrence of CSF rhinorrhea during a mean follow-up of 35.25 months. Of the patients two showed recurrence of CSF rhinorrhea after the first surgery (patient 1 and 3). One patient (patient 3) showed recurrence of CSF rhinorrhea postoperatively after closure with an onlay of DuraGen® and fat. Preoperative imaging before the second surgery showed the same localization of the defect. The most likely cause for recurrence was insufficient exposure of the defect and closure with an overlay only. Another patient (patient 1) had only CTC prior to her first surgery as MRC was not available during that time. No CSF flow was visible during surgery and therefore only broad sealing of the anterior skull base with an overlay only in the suspected area of the leak on CTC could be performed. These cases highlight the importance of precise localization and stable closure of CSF leaks to prevent recurrence and associated complications.

In this case series, the gasket-seal technique appears to provide a stable closure of the defect, which is important in preventing recurrence, perhaps due to a stable closure as autologous bone or alloplastic bone material is countersunk into the defect and provides a watertight seal in combination with the overlay. Different studies have shown reliable results, mainly after endonasal anterior skull base surgery, with recurrence rates of 0–4.3% [12, 22–24]. For closure of most small and medium sized defects of the anterior skull base, the gasket-seal technique is now the method of choice in our departments.

The main limitation of our retrospective case series is the inclusion of only a small number of patients with idiopathic or postoperative CSF leaks. As MRC with CS T1 SPACE sequence is a new technique, the time frame of patient recruitment was limited. Larger studies are necessary to confirm our early results.

## Conclusion

MR cisternography with CS T1 SPACE sequence could be a promising technique for precise localization of CSF leaks, which is important to plan surgical access, increase the success rate and reduce complications. The gasket-seal technique resulted in good closure of the CSF fistula in this case series.

## Abbreviations

BMI: Body mass index
CSF: Cerebrospinal fluid
CS T1 SPACE: T1 SPACE sequence with compressed-sensing method
CT: Computed tomography
CTC: Contrast-enhanced CT cisternography
GCP: Note for guidance on good clinical practise
IIH: Idiopathic intracranial hypertension
MR: Magnetic resonance
MRC: Intrathecal gadolinium-enhanced MR cisternography

## References

[1] Georgalas C, Oostra A, Ahmed S, Castelnuovo P, Dallan I, van Furth W (2021). International consensus statement: spontaneous cerebrospinal fluid rhinorrhea. Int Forum Allergy Rhinol.

[2] Schlosser RJ, Woodworth BA, Wilensky EM, Grady MS, Bolger WE (2006). Spontaneous cerebrospinal fluid leaks: a variant of benign intracranial hypertension. Ann Otol Rhinol Laryngol.

[3] Yang Z, Wang B, Wang C, Liu P (2011). Primary spontaneous cerebrospinal fluid rhinorrhea: a symptom of idiopathic intracranial hypertension?. J Neurosurg.

[4] Lindstrom DR, Toohill RJ, Loehrl TA, Smith TL (2004). Management of cerebrospinal fluid rhinorrhea: the Medical College of Wisconsin experience. Laryngoscope.

[5] Daudia A, Biswas D, Jones NS (2007). Risk of meningitis with cerebrospinal fluid rhinorrhea. Ann Otol Rhinol Laryngol.

[6] Kubik M, Lee S, Snyderman C, Wang E (2017). Neurologic sequelae associated with delayed identification of iatrogenic skull base injury during endoscopic sinus surgery (ESS). Rhinology.

[7] Psaltis AJ, Schlosser RJ, Banks CA, Yawn J, Soler ZM (2012). A systematic review of the endoscopic repair of cerebrospinal fluid leaks. Otolaryngol Head Neck Surg.

[8] Mokri B (1999). Spontaneous cerebrospinal fluid leaks: from intracranial hypotension to cerebrospinal fluid hypovolemia—evolution of a concept. Mayo Clin Proc.

[9] Schievink WI (2006). Spontaneous spinal cerebrospinal fluid leaks and intracranial hypotension. JAMA.

[10] Aydin K, Guven K, Sencer S, Jinkins JR, Minareci O (2004). MRI cisternography with gadolinium-containing contrast medium: its role, advantages and limitations in the investigation of rhinorrhoea. Neuroradiology.

[11] Duman IE, Demerath T, Stadler A, Elsheikh S, Raithel E, Forman C (2021). High-resolution gadolinium-enhanced MR cisternography using compressed-sensing T1 SPACE technique for detection of Intracranial CSF leaks. Ajnr Am J Neuroradiol.

[12] Leng LZ, Brown S, Anand VK, Schwartz TH (2008). “Gasket-seal” watertight closure in minimal-access endoscopic cranial base surgery. Neurosurgery.

[13] Association, D. I. Note for Guidance on Good Clinical Practice (CPMP/ICH/135/95): Declaration of Helsinki and the Belmont Report: Ethical Principles and Guidelines for the Protection of Human Subjects of Research, DIA. 1997.

[14] Algin O, Turkbey B (2013). Intrathecal gadolinium-enhanced MR cisternography: a comprehensive review. AJNR Am J Neuroradiol.

[15] Eljazzar R, Loewenstern J, Dai JB, Shrivastava RK, Iloreta AM (2019). Detection of cerebrospinal fluid leaks: is there a radiologic standard of care? A systematic review. World Neurosurg.

[16] Reddy M, Baugnon K (2017). Imaging of cerebrospinal fluid rhinorrhea and otorrhea. Radiol Clin North Am.

[17] Tali ET, Ercan N, Krumina G, Rudwan M, Mironov A, Zeng QY (2002). Intrathecal gadolinium (gadopentetate dimeglumine) enhanced magnetic resonance myelography and cisternography: results of a multicenter study. Invest Radiol.

[18] Kapoor R, Liu J, Devasenapathy A, Gordin V (2010). Gadolinium encephalopathy after intrathecal gadolinium injection. Pain Phys.

[19] Arlt S, Cepek L, Rustenbeck HH, Prange H, Reimers CD (2007). Gadolinium encephalopathy due to accidental intrathecal administration of gadopentetate dimeglumine. J Neurol.

[20] Tali ET, Ercan N, Kaymaz M, Pasaoglu A, Jinkins JR (2004). Intrathecal gadolinium (gadopentetate dimeglumine)-enhanced MR cisternography used to determine potential communication between the cerebrospinal fluid pathways and intracranial arachnoid cysts. Neuroradiology.

[21] Rupa V, Jasper A, Abraham L, Rajshekhar V (2022). MR findings suggestive of idiopathic intracranial hypertension in 117 patients with spontaneous cerebrospinal fluid rhinorrhea. Neuroradiology.

[22] Garcia-Navarro V, Anand VK, Schwartz TH (2013). Gasket seal closure for extended endonasal endoscopic skull base surgery: efficacy in a large case series. World Neurosurg.

[23] Wessell A, Singh A, Litvack Z (2013). One-piece modified gasket seal technique. J Neurol Surg B Skull Base.

[24] Fraser JF, Nyquist GG, Moore N, Anand VK, Schwartz TH (2010). Endoscopic endonasal minimal access approach to the clivus: case series and technical nuances. Neurosurgery.

